# A New Nomogram and Risk Stratification of Brain Metastasis by Clinical and Inflammatory Parameters in Stage III Small Cell Lung Cancer Without Prophylactic Cranial Irradiation

**DOI:** 10.3389/fonc.2022.882744

**Published:** 2022-07-07

**Authors:** Jianjian Qiu, Dongmei Ke, Yilin Yu, Hancui Lin, Qunhao Zheng, Hui Li, Hongying Zheng, Lingyun Liu, Zhiping Wang, Yahua Wu, Tianxiu Liu, Jiancheng Li

**Affiliations:** Fujian Medical University Cancer Hospital, Fujian Cancer Hospital, Fuzhou, China

**Keywords:** small cell lung cancer, brain metastasis, risk factor, risk stratification, nomogram

## Abstract

**Background:**

This study was conducted to determine risk factors for developing brain metastasis (BM) and to predict brain metastasis free survival (BMFS) and overall survival (OS) by combining several clinical parameters and inflammatory indexes.

**Materials and Methods:**

A nomogram and risk stratification were developed based on multivariate analysis results. The prognostic index (PI) predicting the high risk of BM was calculated by multiplying the weighted factor (β coefficient) with each variable.

**Results:**

Thirty-two of one hundred patients (32.0%) developed BM. Multivariate cox regression analysis revealed that concurrent chemoradiotherapy (CCRT; hazard ratio (HR), 3.356; p = 0.020), monocyte–lymphocyte ratio (MLR; HR, 4.511; p = 0.002), neutrophil–lymphocyte ratio (NLR; HR, 4.023; p = 0.033), and prognostic-nutrition index (PNI; HR, 2.902; p = 0.018) were independent prognostic factors of BMFS. The nomogram has good accuracy in predicting BMFS, and the C-index was 0.73. The ROC curve showed that these risk factors have good discriminant ability. Similarly, tumor location (HR, 1.675; p = 0.035) and MLR (HR, 2.076; p = 0.013) were independent prognostic factors of OS. In the subgroup analysis of OS, the good group had a better prognosis than the other groups. Risk stratification by PI: the high-risk group had worse BMFS than the low-risk group, which also has certain practical significance for clinical practice in OS.

**Conclusion:**

We developed a nomogram and corresponding risk stratification in stage III SCLC patients who developed BM. This model and risk stratification can help clinicians improve patient treatment management and better deliver personalized therapy.

## Introduction

Small cell lung cancer (SCLC) is an aggressive neuroendocrine malignant tumor characterized by rapid doubling time and poor prognosis, with only one-third of patients in limited-stage (1). Chemoradiotherapy (CRT) is the primary treatment of limited-stage SCLC (LS-SCLC) (2, 3). Compared with chemotherapy alone, the multimodality treatment improves survival rates significantly. Although the primary tumor is sensitive to chemotherapy and radiotherapy, local recurrence or metastatic spread is common shortly after treatment (4). The brain is considered a refuge for relapse because the blood–brain barrier blocks the entry of most chemotherapy drugs. Therefore, the brain is a common metastatic site of SCLC, and more than 50% of patients with LS-SCLC still develop intracranial metastasis after completion of CRT (5). Autopsy studies have demonstrated that one-half to two-thirds of patients with SCLC develop brain metastasis (BM) at death (6, 7).

Even though prophylactic cranial irradiation (PCI) reduced the occurrence of BM, the incidence of BM in patients with SCLC remains high. The effect of PCI on the overall survival (OS) of patients with SCLC is still controversial. The randomized trial data showed that PCI reduced the rate of BM from about 60 to 30% and increased the 3-year OS by about 5% (8). Another multicenter and randomized trial result demonstrated that PCI reduced the rate of BM, but failed to improve the OS of the patient (9). However, about 20–40% of patients are diagnosed with BM even after PCI (10). Additionally, PCI can cause discomfort such as dizziness, lethargy, and loss of appetite, and some patients even have serious side effects such as neurocognitive impairment and hormone deregulation (11). These symptoms significantly affect the quality of life of patients, which our clinicians must consider, particularly in SCLC. Besides, not all patients with LS-SCLC receive PCI treatment in reality. A study indicated that patients with BM at the time of initial diagnosis had significantly higher survival than those diagnosed with BM after completion of CRT (12). Therefore, early identification of risk factors related to particular patients with BM would be valuable for therapeutic management and improving SCLC prognosis.

SCLC patients with BM usually have a poor prognosis with a median survival time of 2–14 months (13). It has been proved that the factors correlated with the prognosis of SCLC patients with BM include age, performance status, number of brain lesions, and so on (14). However, few studies have investigated the prognostic impact of inflammation markers in LS-SCLC patients diagnosed with BM after receiving CRT. Inflammatory and immune responses are indispensable to the development and metastasis of tumors (15). For example, several studies have linked increased platelet counts or decreased lymphocyte counts to poor prognosis in lung cancer patients (15, 16). Neutrophil–lymphocyte ratio (NLR) has been identified as a risk factor associated with BM in SCLC (17). Interestingly, the neutrophils, lymphocytes, monocytes, platelets, and albumin could be easily measured during treatment. Therefore, we carried out this study to explore the risk factors of developing BM after initial treatment in patients with stage III LS-SCLC through the inflammatory index and clinical factors.

## Materials and Methods

### Patients and Follow-Up

We reviewed patients with stage III LS-SCLC who received CRT at the Fujian Provincial Cancer Hospital from 2007 to 2019. All patients received a standardized evaluation before antineoplastic treatment, namely, thoracic and abdominal computed tomography (CT) with contrast medium, radionuclide bone scanning, and cranial magnetic resonance imaging (MRI) or CT with contrast medium, or position emission tomography/computerized tomography (PET/CT). The patients were staged according to the TNM classification of the eighth edition of the American Joint Commission on Cancer (AJCC, 8th edition) and the two-stage system based on version 1.2016 of the National Comprehensive Cancer Network Guidelines for SCLC (NCCN2016). Inclusion criteria were (1): pathology and imaging proved LS-SCLC; (2) without BM at first diagnosis; (3) availability of clinical data and peripheral blood cell counts; and (4) receiving CRT. Patients who received PCI were excluded. Finally, 100 stage III LS-SCLC patients were enrolled. The Ethics Committee approved this study at the Fujian Provincial Cancer Hospital.

Patients were generally reexamined at the end of every 2 or 3 cycles of chemotherapy and at the beginning and end of radiotherapy. After completing the initial treatment, patients were usually revisited every three months for the first two years, every six months for 3 to 5 years, and yearly thereafter. However, this follow-up time was not permanently fixed. Thoracic CT or PET/CT images were usually obtained at each follow-up. Brain MRI or CT should be performed at each follow-up despite the variation.

### Chemotherapy and Radiotherapy

All patients received individualized CRT. Chemotherapy regimens included etoposide, paclitaxel, or irinotecan with cisplatin, carboplatin, nedaplatin, or lobaplatin. The median cycle of chemotherapy was five cycles. Thoracic radiotherapy was performed using 3-dimensional conformal radiotherapy (3D-CRT) or intensity-modulated radiotherapy (IMRT). Most of the patients received conventional fractionated radiotherapy (CFRT), while a few patients received hyper-fractionated radiotherapy (HFRT). Individual radiation was performed with CFRT with 42–69 Gy in 20–33 daily fractions or HFRT with 45–48 Gy in 15–16 fractions. Radiotherapy (RT) used a 6MV medical linear accelerator. The gross tumor volume (GTV) included the primary lung tumor and elective or involved lymph nodes. Considering the microscopic spread, the clinical tumor volume (CTV) comprised the GTV with an edge of 5 mm in all directions. On the basis of CTV, the planned tumor volume (PTV) expanded 5–8 mm in all directions.

### Inflammatory and Nutritionl Index

The systemic immune-inflammation index (SII), monocyte–lymphocyte ratio (MLR), neutrophil–lymphocyte ratio (NLR), platelet–lymphocyte ratio (PLR), prognostic-nutrition index (PNI), and platelet–albumin ratio (PAR) were calculated as follows: SII = absolute neutrophil count times absolute platelet count divided by absolute lymphocyte count; MLR = absolute monocyte count divided by absolute lymphocyte count. NLR = absolute neutrophil count divided by absolute lymphocyte count. PLR = absolute platelet count divided by absolute lymphocyte count. PNI = serum albumin level plus five times the absolute lymphocyte count. PAR = absolute platelet count divided by serum albumin level. These inflammatory indexes were calculated using the blood biochemical data collected within five days before therapy.

### Developing Prognostic Index for Brain Metastasis

The prognostic index (PI) predicting a high risk of brain metastases was calculated in the study population using the Cox regression model. Each independent prognostic factor is multiplied by a β coefficient. Then, the prognostic index was generated by summing. Finally, the study population was divided into the high-risk and low-risk groups according to the risk score calculated from the PI.

### Endpoint*s*

The primary endpoint of interest was brain metastasis-free survival (BMFS), defined as the period from pathological diagnosis to the date of discovery of BM. Overall survival (OS) was calculated as the time from pathological diagnosis to death or the last follow-up. The median follow-up time was 35.8 months (9.2–153.8 months).

### Statistical Analysis

Statistical analyses were performed using SPSS software (version 25.0) and R software (version 4.0.2). The optimal cut-off values of time from initial treatment to radiotherapy, cycle of chemotherapy before radiotherapy, cycle of chemotherapy before BM, RT dose, SII, MLR, NLR, PLR, PNI, PAR, and PI were calculated using the X-tile software (version 3.6.1), which is essential for generating the best cut-off point with the minimum p-value. The Chi-squared tests or Fisher’s exact tests compared the categorical variables. The plot survival curve was drawn using the Kaplan–Meier method, and the log-rank test was used to compare the difference in the survival curve. Variables with p <0.2 in univariate analysis were incorporated into multivariate Cox regression analysis. Multivariate Cox regression analysis was used to determine independent risk factors related to survival. Based on BMFS multivariate analysis, only the factors with a p-value of <0.05 were included in the nomogram. The performance of the nomogram was evaluated by the calibration curve (with 1,000 bootstrap resamples) and the C-index. The larger the C-index, the more accurate the prediction. The clinical value of risk factors for BM was analyzed by receiver operating characteristic (ROC) curves. All tests were double-tailed, and a p-value less than 0.05 was considered statistically significant.

## Results

### Patient Characteristics

The medical records of 100 patients with stage III SCLC were collected and analyzed. **Table 1** summarizes the clinical characteristics of the patients. Among them, 93 (93.0%) were men, 49 (49.0%) were ≥60 years old, 57 (57.0%) had a history of smoking, 60 (60.0%) were located in the right lung, 47 (47.0%) received concurrent chemoradiotherapy, and 32 (32.0%) had BM. The best cut-off points of time from initial treatment to radiotherapy, cycle of chemotherapy before radiotherapy, cycle of chemotherapy before BM, RT dose, SII, MLR, NLR, PLR, PNI, PAR, and PI were 3, 4, 5, 52.5, 937.3, 0.12, 3.23, 97.3, 51.4, 4.38, and 2.46, respectively.

**Table 1:** Baseline characteristics of patients.

Variable		Total	Percentage
Gender			
	Male	93	93.0%
	Female	7	7.0%
Age (years)			
	<60	51	51.0%
	≥60	49	49.0%
Smoke			
	No	43	43.0%
	Yes	57	57.0%
Tumor location			
	Left lung	40	40.0%
	Right lung	60	60.0%
BM			
	No	68	68.0%
	Yes	32	32.0%
Time from initial treatment to radiotherapy (months)			
	≤3	67	67.0%
	>3	33	33.0%
Cycle of chemotherapy before radiotherapy			
	≤4	83	83.0%
	>4	17	17.0%
Cycle of chemotherapy before BM			
	≤5	51	51.0%%
	>5	49	49.0%%
CCRT			
	No	53	53.0%
	Yes	47	47.0%
RT dose (Gy)			
	≤52.5	28	28.0%
	>52.5	72	72.0%
T			
	T1	13	13.0%
	T2–4	67	67.0%
	Tx	20	20.0%
N			
	N0–1	5	5.0%
	N2–3	95	95.0%
SII			
	≤937.3	89	89.0%
	>937.3	11	11.0%
MLR			
	≤0.12	23	23.0%
	>0.12	77	77.0%
NLR			
	≤3.23	79	79.0%
	>3.23	21	21.0%
PLR			
	≤97.3	29	29.0%
	>97.3	71	71.0%
PNI			
	≤51.4	75	75.0%
	>51.4	25	25.0%
PAR			
	≤4.38	26	26.0%
	>4.38	74	74.0%

BM, brain metastasis; CCRT, concurrent chemoradiotherapy; RT, radiotherapy; SII, systemic immune-inflammation index; MLR, monocyte–lymphocyte ratio; NLR, neutrophil– lymphocyte ratio; PLR, platelet–lymphocyte radio; PNI, prognostic-nutrition index; PAR, platelet–albumin ratio.

### Prognostic Factors for BMFS

The median time of BMFS was 27.6 months. **Table 2** shows the results of the univariate and multivariate Cox analyses of BMFS. Univariate analyses showed that time from initial treatment to radiotherapy, the cycle of chemotherapy before radiotherapy, the cycle of chemotherapy before BM, concurrent chemotherapy (CCRT), RT dose, MLR, NLR, PLR, PNI, and PAR were associated with BMFS. Multivariate analyses showed that CCRT [hazard ratio (HR) = 3.356, p = 0.020], MLR (HR = 4.511, p = 0.002), NLR (HR = 4.023, p = 0.033), and PNI (HR = 2.902, p = 0.018) were independent prognosis factors of BMFS.

**Table 2:** Log-rank tests and Cox regression analysis of factors associated with BMFS.

Parameters	Univariate Analysis	Multivariate Analysis		
	P	HR	95% CI	P
Gender				
Female vs Male	0.843			
Age (years)				
≥60 vs <60	0.389			
Smoke				
Yes vs No	0.510			
Tumor location				
Left lung vs Right lung	0.534			
Time from initial treatment to radiotherapy (months)				
>3 vs ≤3	0.064	1.113	0.372–3.327	0.848
Cycle of chemotherapy before radiotherapy				
>4 vs ≤4	0.011	2.655	0.874–8.064	0.085
Cycle of chemotherapy before BM				
>5 vs ≤5	0.117	1.219	0.513–2.894	0.654
CCRT				
No vs Yes	0.061	3.356	1.209–9.319	0.020
RT dose (Gy)				
≤52.5 vs >52.5	0.085	1.469	0.662–3.261	0.345
T				
Tx vs T2–4 vs T1	0.931			
N				
N2–3 vs N0–1	0.483			
SII				
>937.3 vs ≤937.3	0.020	1.337	0.354–5.049	0.669
MLR				
≤0.12 vs >0.12	0.013	4.511	1.718–11.844	0.002
NLR				
>3.23 vs ≤3.23	0.021	4.023	1.120–14.457	0.033
PLR				
≤97.3 vs >97.3	0.145	2.043	0.843–4.955	0.114
PNI				
>51.4 vs ≤51.4	0.103	2.902	1.197–7.031	0.018
PAR				
≤4.38 vs >4.38	0.134	1.206	0.515–2.820	0.666

BMFS, brain metastasis free survival; HR, hazards ratio; CI, 95% confidence interval; BM, brain metastasis; CCRT, concurrent chemoradiotherapy; RT, radiotherapy; SII, systemic immune-inflammation index; MLR, monocyte–lymphocyte ratio; NLR, neutrophil–lymphocyte ratio; PLR, platelet–lymphocyte radio; PNI, prognostic-nutrition index; PAR, platelet–albumin ratio.

### Risk Prediction Model for BMFS

**Figure 1:**
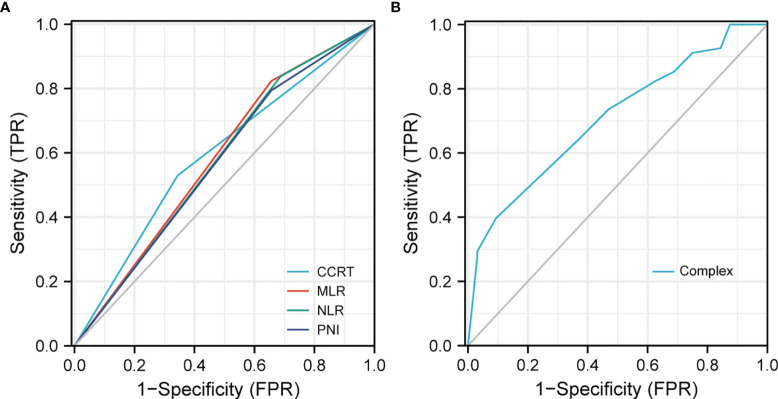
ROC to predict BM. **(A)** The area under of the curve (AUC) of CCRT, MLR, NLR, and PNI was 0.593, 0.584, 0.575, and 0.569. **(B)** AUC of the complex (CCRT, MLR, NLR, and PNI) was 0.708.

**Figure 2:**
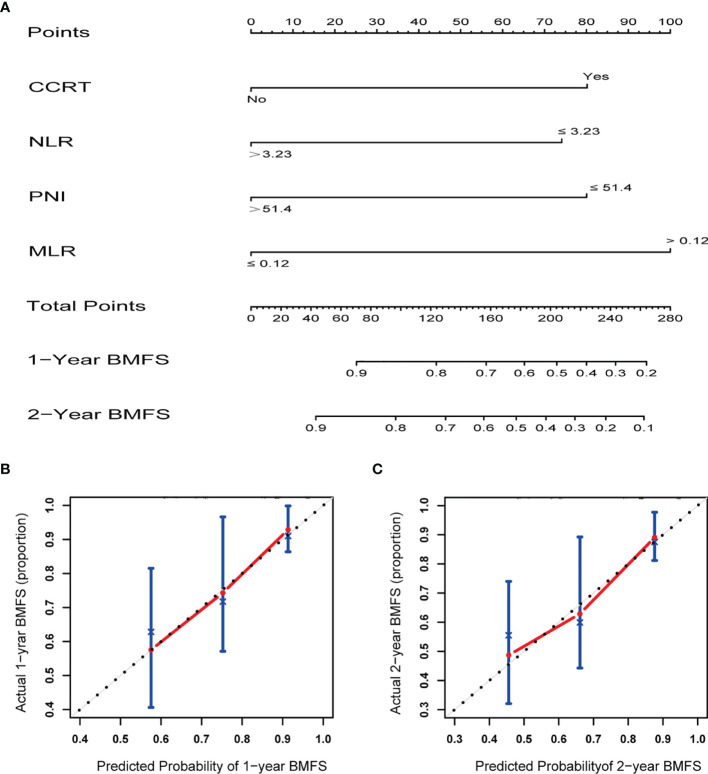
**(A)** Nomogram for prediction 1- and 2-year brain metastasis free survival of stage III small cell lung cancer. **(B)** Calibration curves demonstrating the probability of 1-year BMFS between the prediction and the actual observation. **(C)** Calibration curves demonstrating the probability of 2-year BMFS between the prediction and the actual observation. X-axis represents the nomogram predicted probability, and Y-axis represents the actual observation.

**Figure 3:**
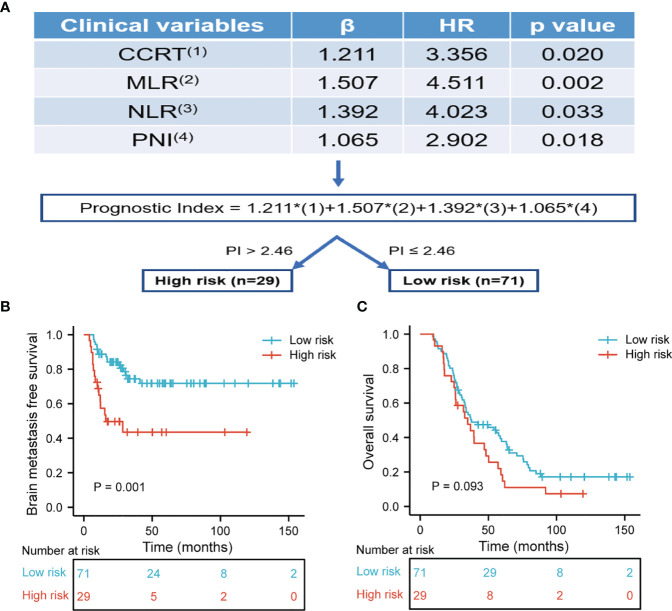
**(A)** The risk prediction model of BM. The prognostic index for predicting BMFS was calculated by multiplying the weighted factor (β coefficient) with four statistically significant variables. Each factor is one for existence and zero in the absence. The prognostic index was divided into high-risk and low-risk subset with a boundary of 2.46. **(B)** Brain metastasis free survival was risk stratification according to prognostic index. The high-risk group had poor BMFS than the low-risk group (p = 0.001). **(C)** Overall survival was risk stratification according to prognostic index. The high-risk group had poor OS than the low-risk group, but it was not statistically significant (p = 0.093).

Based on the results of BMFS multivariate analysis, the variables included in the nomogram were as follows: CCRT, MLR, NLR, and PNI. The ROC of CCRT, MLR, NLR, PNI, and complex (CCRT, MLR, NLR, and PNI) are shown in **Figures 1A, B**. The complex’s area under the curve (AUC) was 0.708, which was higher than each independent risk factor. The nomogram results were reported as 1-year and 2-year BMFS (**Figure 2A**). The C-index of the nomogram was 0.73. The 1-year and 2-year BMFS calibration curves displayed favorable consistency between the actual observations and predicted values (**Figures 2B, C**). PI for predicting BM was obtained by multiplying the weighted factor (β coefficient) with each variable (**Figure 3A**). The patients with a PI higher than 2.46 were in the high-risk groups (n = 29), while those with a PI lower than 2.46 were in the low-risk group (n = 71). The high-risk group had a poorer BMFS than the low-risk group (**Figure 3B**). The high-risk group had poorer OS than the low-risk group, but it was not statistically significant (p = 0.093) (**Figure 3C**).

### Prognostic Factors for OS

**Figure 4:**
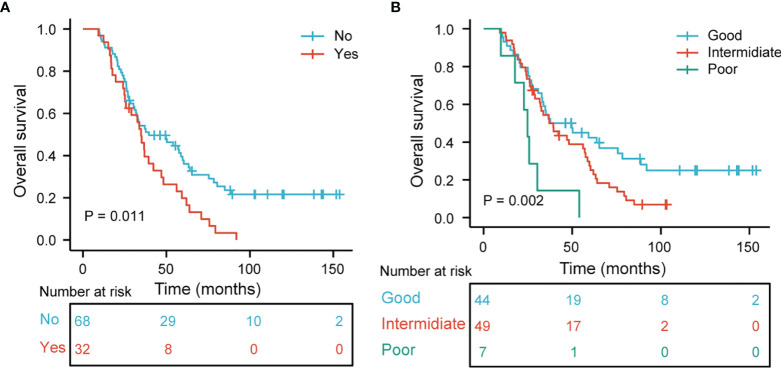
**(A)** Kaplan–Meier curves and log-ranks tests of overall survival in stage III SCLC patients according to brain metastasis. **(B)** Overall survival subgroup analysis according to tumor location and MLR (p = 0.002).

The median OS was 35.8 months. **Table 3** shows the results of univariate and multivariate Cox analyses of OS. In **Table 3**, univariate analyses revealed that tumor location, BM (**Figure 4A**), time from initial treatment to radiotherapy, CCRT, MLR, NLR, and PLR were potential risk factors for OS. On multivariate analysis, tumor location (HR = 1.675, p = 0.035) and MLR (HR = 2.076, p = 0.013) were independently related to worse prognosis. The chemotherapy regimen showed no statistical difference in OS.

**Table 3:** Log-rank tests and Cox regression analysis of factors associated with OS.

Parameters	Univariate Analysis	Multivariate Analysis		
	P	HR	95% CI	P
Gender				
Female vs Male	0.563			
Age (years)				
≥60 vs <60	0.339			
Smoke				
Yes vs No	0.758			
Tumor location				
Left lung vs Right lung	0.027	1.675	1.036–2.707	0.035
BM				
Yes vs No	0.011	1.266	0.743–2.156	0.386
Time from initial treatment to radiotherapy (months)				
>3 vs ≤3	0.076	1.249	0.719–2.171	0.431
Cycle of chemotherapy before radiotherapy				
>4 vs ≤4	0.254			
Cycle of chemotherapy before BM				
>5 vs ≤5	0.715			
CCRT				
No vs Yes	0.037	1.450	0.860–2.444	0.163
RT dose (Gy)				
≤52.5 vs >52.5	0.791			
T				
Tx vs T2–4 vs T1	0.261			
N				
N2–3 vs N0–1	0.551			
SII				
>937.3 vs ≤937.3	0.025	2.212	0.872–5.616	0.095
MLR				
≤0.12 vs >0.12	0.106	2.076	1.168–3.689	0.013
NLR				
>3.23 vs ≤3.23	0.132	1.063	0.482–2.346	0.880
PLR				
≤97.3 vs >97.3	0.162	1.311	0.765–2.249	0.324
PNI				
>51.4 vs ≤51.4	0.966			
PAR				
≤4.38 vs >4.38	0.619			

OS, overall survival; HR, hazards ratio; CI, 95% confidence interval; BM, brain metastasis; CCRT, concurrent chemoradiotherapy; RT, radiotherapy; SII, systemic immune-inflammation index; MLR, monocyte–lymphocyte ratio; NLR, neutrophil–lymphocyte ratio; PLR, platelet–lymphocyte radio; PNI, prognostic-nutrition index; PAR, platelet–albumin ratio.

### OS Stratified by Tumor Location and MLR

We further analyzed the relationship between tumor location and MLR to OS. Patients with the right lung and MLR >0.12 were considered a good group. Patients with a left lung and MLR ≤0.12 were considered the poor group. The remaining combinations were considered the intermediate group. **Figure 4B** demonstrates that the good group, rather than the intermediate and poor groups, had a better OS (p = 0.002 for all).

## Discussion

As an invasive tumor, SCLCs are prominent with high prevalence rates of BM. The current research results have reached a consensus that PCI can reduce the incidence of BM, but whether it can improve the prognosis of patients remains controversial (9, 10). Moreover, a considerable proportion of LS-SCLC patients do not receive PCI treatment because they are worried about side effects. Additionally, the changes in chronic neurotoxicity and quality of life caused by PCI have been reported (18). A retrospective study demonstrated that 44.7% of eligible LS-SCLC patients were without PCI, and the most frequent reason for the recorded omission of PCI was that patients refused to undergo PCI because of potential toxicity (19). The outcomes of SCLC patients with BM are generally poor. If PCI is used to treat specific patients prone to BM, it will improve the prognosis. Therefore, it is helpful for clinicians to improve the treatment strategy of SCLC by identifying the risk of BM in patients who do not receive PCI.

There have been limited studies on the treatment and risk factors in patients with LS-SCLC and BM. There are no reported risk factors for stage III LS-SCLC patients who developed BM after receiving chemoradiotherapy without PCI. In this study, we established and verified a nomogram and risk stratification for predicting the prognosis of stage III LS-SCLC patients who develop BM. The predictors included CCRT, MLR, NLR, and PNI. Different statistical methods verified the model. PI divided the patients into two subgroups, and the high-risk group had worse BMFS than the low-risk group, which also has certain significance for clinical practice in OS. Therefore, this result is helpful for clinicians to improve the treatment management of patients in the high-risk group, such as elective PCI for this group. Besides, the results showed that tumor location and MLR were correlated with OS. In the subgroup analysis of OS, the good group had a better prognosis than the other groups.

CCRT is the main treatment pattern for patients with LS-SCLC. Although CCRT can improve the prognosis, its potential side effects (myelosuppression and radiation-related inflammation responses) cannot be ignored by every clinician (20, 21). Therefore, according to the nutritional status and treatment tolerance condition of each patient, about 47% of the patients in this study received CCRT, and 53% received sequential chemoradiotherapy. Additionally, previous studies have demonstrated that CCRT has better progression-free survival than sequential chemoradiotherapy (SCRT) in SCLC or other cancers, reducing the risk of distant metastasis (22–24). The fundamental principle for uniting chemotherapy and radiotherapy is to integrate the benefits of chemotherapy in reducing the risk of metastatic disease with the benefits of radiotherapy in local-regional control. Interestingly, CCRT has better BMFS than SCRT in our study. CCRT usually has a better response rate than SCRT, chemotherapy, or radiotherapy alone. Farkhad et al. investigated the association between the treatment response of primary tumor and BMFS, and results showed that the incidence of BM in poor responders to chemoradiotherapy (stable disease/local progression) was significantly increased compared with partial and complete responders (25). Clinical trials have demonstrated that distant control can be corrected by improving local treatment (22, 26). Importantly, patients treated with early CCRT had a lower incidence of subsequent BM (27, 28). Therefore, CCRT can directly improve the therapeutic effect of the primary tumor and indirectly reduce the incidence of distant metastasis in patients.

Inflammation and immune response play a crucial role in tumor development, immune surveillance, and therapeutic response (29–31). Some inflammatory indexes associated with blood components promote cancer progression, such as NLR and platelets (32, 33). Furthermore, two previous studies have reported that high NLR was associated with BM in SCLC patients without PCI (17, 34). This conclusion is consistent with our results that high NLR and low MLR have poor BMFS. The plausible explanation is that the tumor response mediated by increased neutrophils and decreased monocytes may play a role. From a clinical perspective, cancer therapy can trigger a significant tumor-associated inflammatory response. For example, chemoradiotherapy can cause massive necrosis of the surrounding tissues and cancer cells, leading to an inflammatory response. Treatment-induced inflammation may have a tumor-promoting effect (35). Non-steroidal anti-inflammatory drugs reduce cancer risk (36) and may prevent tumor metastasis (37).

Our study found that MLR and tumor location were closely related to OS. The myeloid lineage cells (monocytes, macrophages, and neutrophils) gradually accumulate in tumors, where they construct an inflammatory tumor microenvironment (31). It has been suggested that tumor-associated macrophages, which result from monocytic precursors, play a crucial role in the inflammatory microenvironment of cancer progression (38). In many studies (39, 40), MLR represents relative levels of monocytes and lymphocytes in the peripheral blood, and its prognostic value has been observed. Although some studies have shown that tumor location impacts prognosis, different studies have different classification standards for tumor location. Hyun et al. demonstrated that upper lobe tumors were related to better survival than lower lobe tumors (41). Yang et al. reported that the primary tumor site had a significant influence on lung adenocarcinoma prognosis, with the main bronchus site associated with poorer prognosis and more lymph node involvement (42). Li et al. showed that the left lung had a poorer outcome than the right lung, but the difference was statistically insignificant (p = 0.071) (43). Interestingly, this is consistent with our finding that the left lung has a worse prognosis than the right lung. However, the biological mechanisms underlying the relationship between tumor location and prognosis remain unclear. There is an association between the lung tumor site and lymph node metastasis. Tsuguo et al. examined lymph node metastasis in 1,815 patients with lung cancer. They found that lymph node metastasis in patients with left lung cancer was significantly higher than in patients with right lung cancer (44). It is well known that the higher the lymph node metastasis rate of lung cancer, the worse the prognosis. Another theory is that the same treatment could have different effects depending on the location of the tumor. The left lung is anatomically adjacent to the heart, so clinicians have to consider the effects of dose and other factors on the heart when designing and delineating target volume. Further multicenter, prospective studies are needed to confirm our results and elucidate the underlying biological mechanisms.

The limitations of this study must be acknowledged. First, this was a retrospective study of one institution, which had its limitations, such as inevitable selection bias. Secondly, the number of patients enrolled in this study was relatively small. Thirdly, our study was limited to patients with stage III SCLC. Since patients with stage III SCLC account for most of the data, we excluded patients in other stages to reduce the differences between groups caused by staging. Whether other stages can reach the same conclusion is debatable. Further validation is required in a large cohort of patients. Additionally, some known prognostic factors of LS-SCLC, such as tumor markers and tumor size, were not incorporated into the study. Despite these limitations, this study first developed a nomogram model to predict the development of BM in stage III SCLC patients.

## Conclusion

We developed a nomogram and corresponding risk stratification for predicting BMFS in stage III SCLC patients. Various statistical methods to prove that the model had satisfactory performance. According to the risk stratification system, the high-risk group had a worse BMFS than the low-risk group, which also has certain practical significance for clinical practice in OS. This model and risk stratification can help clinicians improve patient treatment management and better deliver personalized therapy. Of course, further studies must confirm this model and the prognostic index for BMFS in stage III SCLC.

## Data Availability Statement

The data that support the findings of this study are available from the corresponding author upon reasonable request.

## Author Contributions

JL and JQ designed this study. JQ and DK contributed to the data collection. HaL, HuL, and YY analyzed the data. JL supervised the study. JQ, DK, HZ, HaL, YY, QZ, LL, HuL, YW, ZW, and TL wrote the manuscript. All authors listed have made a substantial, direct, and intellectual contribution to the work and approved it for publication.

## Funding

The project was supported by the National Clinical Key Specialty Construction Program and Fujian provincial Clinical Research Center for Cancer Radiotherapy and Immunotherapy (Grant number 2020Y2012).

## Conflict of Interest

The authors declare that the research was conducted in the absence of any commercial or financial relationships that could be construed as a potential conflict of interest.

## Publisher’s Note

All claims expressed in this article are solely those of the authors and do not necessarily represent those of their affiliated organizations, or those of the publisher, the editors and the reviewers. Any product that may be evaluated in this article, or claim that may be made by its manufacturer, is not guaranteed or endorsed by the publisher.

## Abbreviations

SCLC, small cell lung cancer; CRT, chemotherapy; LS-SCLC, limited-stage small cell lung cancer; TRT, thoracic radiotherapy; BM, brain metastasis; PCI, prophylactic cranial irradiation (PCI); OS, overall survival; NLR, neutrophil-lymphocyte ratio; PLR, platelet-lymphocyte ratio. CT, computed tomography; MRI, magnetic resonance imaging (MRI); PET/CT, position emission tomography/computerized tomography; AJCC, American joint commission on cancer; NCCN, national comprehensive cancer network guidelines; 3D-CRT, 3-dimensional conformal radiotherapy; IMRT, intensity-modulated radiotherapy; CFRT, conventional fractionated radiotherapy; HFRT, hyper-fractionated radiotherapy; RT, radiotherapy; GTV, gross tumor volume; CTV, clinical tumor volume; PTV, planned tumor volume; SII, systemic immune-inflammation index; MLR, monocyte-lymphocyte ratio; PNI, prognostic-nutrition index; PAR, platelets-albumin ratio; BMFS, brain metastasis free survival; PI, prognostic index; ROC, receiver operating characteristic; CCRT, concurrent chemotherapy; AUC, the area under the curve; SCRT, sequential chemoradiotherapy.
